# Tailored cell sheet engineering using microstereolithography and electrochemical cell transfer

**DOI:** 10.1038/s41598-019-46801-9

**Published:** 2019-07-18

**Authors:** Yuka Kobayashi, Christopher E. J. Cordonier, Yohei Noda, Fuminori Nagase, Junko Enomoto, Tatsuto Kageyama, Hideo Honma, Shoji Maruo, Junji Fukuda

**Affiliations:** 1 Faculty of Engineering, Yokohama National University, 79-5 Tokiwadai, Hodogaya-ku, Yokohama 240-8501 Japan; 2 Faculty of Engineering, Kanto Gakuin University, 1162-2 Ogikubo, Odawara, 250-0022 Japan

**Keywords:** Regenerative medicine, Tissue engineering, Biomaterials - cells, Cell delivery

## Abstract

Postoperative adhesion and occlusion remain a serious issue associated with various surgeries, including endoscopic surgery, in which proliferated fibrous tissues stick to adjacent tissues and often cause severe complications. Cell sheet engineering has emerged as an effective approach not only for cell transplantation but also for the treatment of postoperative adhesion and occlusion. However, as the tissues in the body, such as middle ear and small intestine, and typical operative sites are non-flat and spatially complicated, tailored cell sheets with three-dimensional (3D) configurations may lead to widespread use of this approach. In the present study, we used microstereolithography, biocompatible gold plating, and electrochemical cell detachment to achieve this purpose. Various objects with dimensions ranging from millimeter- to micrometer-scale were fabricated with photocurable resin using lab-made equipment for microstereolithography. To coat the fabricated objects with a thin gold layer, conventional cyanide-based gold plating was unusable because it severely damaged almost all cells. Electroless non-cyanide gold plating we prepared was cytocompatible and suitable for electrochemical cell detachment. Cell sheets on the gold-plated substrate could be directly transplanted into a mouse intraperitoneally using electrochemical cell detachment. We further demonstrated that cell sheets grown on gold-coated 3D objects were rapidly detached along with the desorption of electroactive-oligopeptide monolayer and transferred to a surrounding hydrogel. This approach may provide a promising strategy to prepare and directly transplant tailor-made cell sheets with suitable configurations.

## Introduction

Endoscopic surgery has been widely recognized as a less invasive approach than conventional open surgery. However, postoperative inflammation and coalescence are still major concerns^1^. Recently, it was reported that such complications can be alleviated by placing cell sheets on operative sites after surgery^2,3^. Cell sheet engineering was proposed more than a decade ago and has demonstrated successful outcomes in clinical trials for treatment of the esophagus, periodontal tissue, heart, and cornea^4,5^. Esophageal stenosis following endoscopic submucosal resection for esophageal cancer was successfully cured using autologous mucosal epithelial cell sheets^6^. This approach has led to mature epithelialization and no attacks in patients after endoscopic submucosal dissection^7^. In cell sheet engineering, culture substrates modified with poly(N-isopropyl acrylamide), a thermo-responsive polymer, have been used. The surface properties of the substrates can be dynamically modulated from cell-adhesive to cell-nonadhesive using temperature changes during cell culturing. A two-dimensionally connected cell sheet was prepared and collected from the substrate solely by lowering the temperature to 20 °C. This approach is sophisticated and commercialized, but has potential drawbacks in that it takes a relatively long time (40–60 min) under exposure to a non-physiological temperature to detach the cell sheet and the cell sheet readily shrinks and collapses after detachment^8,9^. To address the latter issue, a polymer supporter was used to hold and transplant the detached cell sheet while maintaining its shape^7^. However, considering the necessity of the relatively long-term low-temperature processes for detachment, it may be challenging to directly transplant a cell sheet from the substrate into the body using this approach. This potentially limits widespread use of this approach because only flat cell sheets are available while surgical sites are typically non-flat, three-dimensional (3D), and complicated.

**Figure 1:**
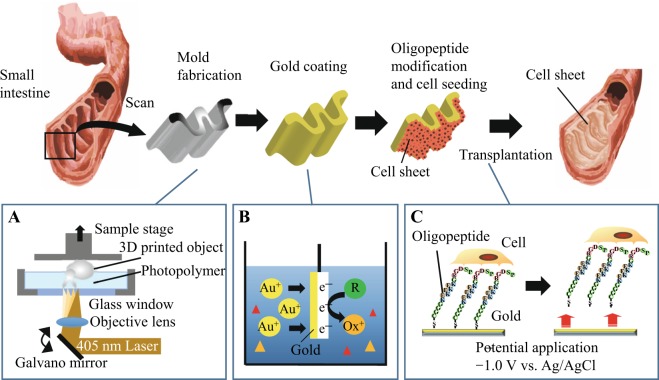
Strategies for transplanting tailor-made cell sheets using electrochemical cell detachment. (**A**) Fabrication of a 3D mold corresponding to a surgery site using microstereolithography. (**B**) Electroless gold plating onto the fabricated mold. (**C**) Direct transplantation using electrochemical cell detachment. A cell sheet was detached and transferred along with electrochemical desorption of the oligopeptide layer.

Other external stimuli, such as optical, electrical, and magnetic stimuli, have been used to trigger the detachment of cells under a culture condition^10^. Among them, we examined an electrical approach in the present study because it may be better suited than others in terms of uniform and rapid cell sheet detachment from 3D molds. In our previous study, cell sheets were rapidly and noninvasively detached from a gold surface using specific electrochemical reactions^11^. In this approach, a zwitterionic oligopeptide, CGGGKEKEKEKGRGDSP, was designed to modify a gold surface^12^. The cysteine (C) residue at the terminal contains a thiol group that spontaneously formed a gold-thiolate (Au-S) bond on the gold surface. The thrice-repeated alternant lysine (K) and glutamic acid (E) residues induced a closely packed oligopeptide layer via electrostatic intermolecular interactions, rendering the modified surface cell-repulsive^13^. By adding the GRGDSP domain at the terminal, the modified surface promoted integrin-mediated cell adhesion via the oligopeptide layer while preventing direct contact of cells with the gold surface, which was important when detaching cells from the surface by electrical stimulus^14^. By applying an electrical potential, the Au-S bond was cleaved and followed by desorption of the oligopeptide layer, by which single cells and cell sheets were detached from the gold surface within 5–10 min^15^. This approach was applicable not only for a flat surface, but also for the cylindrical needles used to fabricate microchannels enveloped with vascular endothelial cells in a hydrogel^16–20^. In this study, we examined whether this approach could be used to prepare tailor-made cell sheets with 3D configurations and transplant them directly into the body (Fig. 1). To this end, we employed microstereolithography, which is applicable for fabricating any 3D mold based on structural information obtained using computer tomography and magnetic resonance imaging. We examined gold-plating methods suitable for coating entire surfaces of 3D molds and then transplanted the cell sheets covering the surfaces using the potential application. We believe this approach could expand the scope of cell sheet engineering applications.

## Results and Discussion

### Cytocompatible gold plating

**Figure 2:**
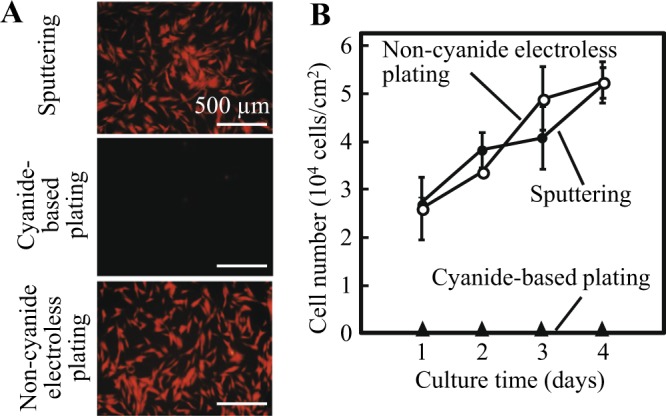
Cell adhesion and growth on gold-plated substrates. (**A**) Fluorescent images of RFP-HNDFs on gold substrates prepared by sputtering, cyanide electrolytic gold plating, and non-cyanide electroless gold plating at 1 d post-culture. (**B**) Proliferation of cells on different gold substrates. The error bars indicate standard deviations calculated from 3 independent experiments.

To apply electrochemical cell detachment to a complicated configuration fabricated using microstereolithography (Fig. 1), it is necessary to find an appropriate approach for the preparation of a stable gold layer on a photocurable resin. In our previous study, gold substrates were prepared by sputter-coating^21^, but this may be unsuitable for this purpose because a sputter-coated gold layer is not stable on a resin and gold grains fly straight in a sputter chamber and accumulate only on one side of a material. Thus, we investigated gold plating in the present study because of its isotropic aspect. Gold plating has been used for commercial medical apparatus such as surgical scissors and tweezers. We initially examined conventional gold plating in collaboration with a medical plating company. However, no cells attached to the surface prepared with this gold plating (Fig. 2A,B, cyanide-based plating). We investigated several possible factors responsible for this phenomenon, including fine surface structures and plating bath components (details not shown, due to confidential corporate information), and eventually determined that a little cyanide remained in the gold layer, which seriously damaged the cells. This was determined by observing the cells attached to the gold surface only after treatment with a strong reductant, NaBH_4_ (data not shown). To avoid this problem and perform gold coating in a single step, we proposed electroless gold plating using tiopronin gold complex instead of the conventional gold cyanide (Table 1). As tiopronin has been used for the treatment of diseases associated with cysteine disulfide, such as cystinuria, this chemical is approved to be biocompatible^22,23^. The tiopronin-based electroless gold plating provided a stable and thin gold layer on the photocurable resin plate, on which cells attached and vigorously grew comparable to the sputter-coated gold surface (Fig. 2A,B). Based on the results, non-cyanide electroless gold plating was used in the following experiments.

**Table 1:** Compositions of electroless gold-plating bath.

Components	Concentration
3-Mercapto-1, 2, 4-triazole	5 g/L
*N*,*N*,*N*′,*N*′-Ethylenediamine tetrakis (methylenephosphonic acid)	5 g/L
Tripotassium citrate monohydrate	15 g/L
Tripotassium phosphate	15 g/L
Polyethylene glycol 1,000	100 mg/L
Pyridine-3-sulfonic Acid	5 g/L
L(+)-Ascorbic acid	18 g/L
RSG2 syrup (TPN-Au, Au ca. 100g/L)	20 ml/L

pH was adjusted to 5.99–6.01 using H_2_SO_4_ and KOH.

### Electrochemical detachment of single cells and cell sheets

**Figure 3:**
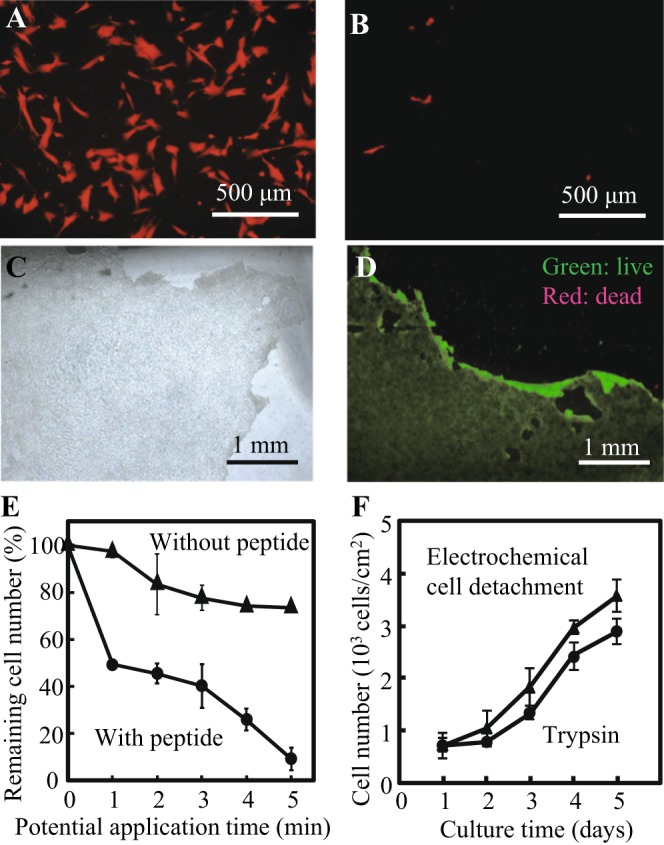
Electrochemical cell detachment from gold substrates prepared with electroless gold plating. (**A**,**B**) Fluorescent images of RFP-HNDFs on the gold substrates modified with oligopeptide before (**A**) and after (**B**) potential application. (**C**) Phase-contrast microscopic image of detached NB1RGB cell sheet. (**D**) Live/dead staining of cells in the detached cell sheet. The cytoplasm of viable cells is green, whereas the nuclei of dead cells are red. (**E**) Changes in the number of cells remaining on the gold substrates after potential application with/without oligopeptide modification. The error bars indicate standard deviations calculated from 3 independent experiments. (**F**) Proliferation of electrochemically detached cells on a conventional culture dish. Cells were collected using potential application for 5 min or trypsin treatment and then seeded on a conventional culture dish. The number of cells were counted on fluorescent microscopic images. The error bars indicate standard deviations calculated from 3 independent experiments.

The oligopeptide was designed to chemically adsorb on a gold surface via gold-thiolate bonding^12^. The adsorbed amount of the oligopeptide on the gold surface prepared with non-cyanide electroless plating was estimated via quartz crystal microbalance (QCM) measurement. This is important because dense molecular layer formation is responsible for reliable electrochemical detachment of cells^13^. The adsorbed amount was 189 ng/cm^2^, which indicates that the distance between neighboring oligopeptides was virtually 1.4 nm given that the oligopeptide aligns in the tetragonal configuration on the surface. This is equivalent to that on the sputter-coated surface (1.3 nm), where a dense self-assembled monolayer formation was suggested^12^. The gold-thiolate bond can be cleaved by applying a negative electrical potential. Our previous study demonstrated that the application of −1.0 V with respect to an Ag/AgCl reference electrode was sufficient to detach cells along with the electrochemical desorption of the oligopeptide layer from a sputter-coated gold surface^12^. In the present study, the same examination was conducted on the gold-plated substrate. As shown in Fig. 3A,B, almost all cells were detached by the potential application. The quantitative analysis revealed that cells were detached up to 20% even without oligopeptide modification, but ~95% of cells were detached with the oligopeptide modification after 5 min of potential application (Fig. 3E). The detached cells were reseeded on a conventional culture dish and subsequently proliferated vigorously. The results were comparable to trypsin-treated cells after at least 5 d of culture (Fig. 3F). These results indicate that the gold layer prepared with electroless gold plating can be used for cell culture and the electrochemical detachment of cells as previously shown with sputter-coated gold surface.

We further examined the gold-plated surface on the detachment of cell sheets. NB1RGB cells were seeded on a gold-coated plate modified with oligopeptide and grown to reach confluence and form a two-dimensionally connected cell layer for 7 d. After a few drops of collagen were added to the cell layer and gelated, an electrical potential was applied to the gold surface for 10 min and the collagen gel was peeled from the surface, resulting in transfer of the cell sheet from the gold surface to the collagen gel (Fig. 3C). Live/dead staining shows that almost all the cells were viable in the detached cell sheet (Fig. 3D). This electrochemical cell detachment was rapid compared to that with the thermo-responsive surface, which requires 40–60 min for cell detachment^9^. Rapid cell sheet detachment may be important not only for minimizing the exposure of cells to non-physiological conditions, such as low temperature, but also for when a cell sheet is directly transplanted into the body.

### Direct transplantation of cell sheets to mice

**Figure 4:**
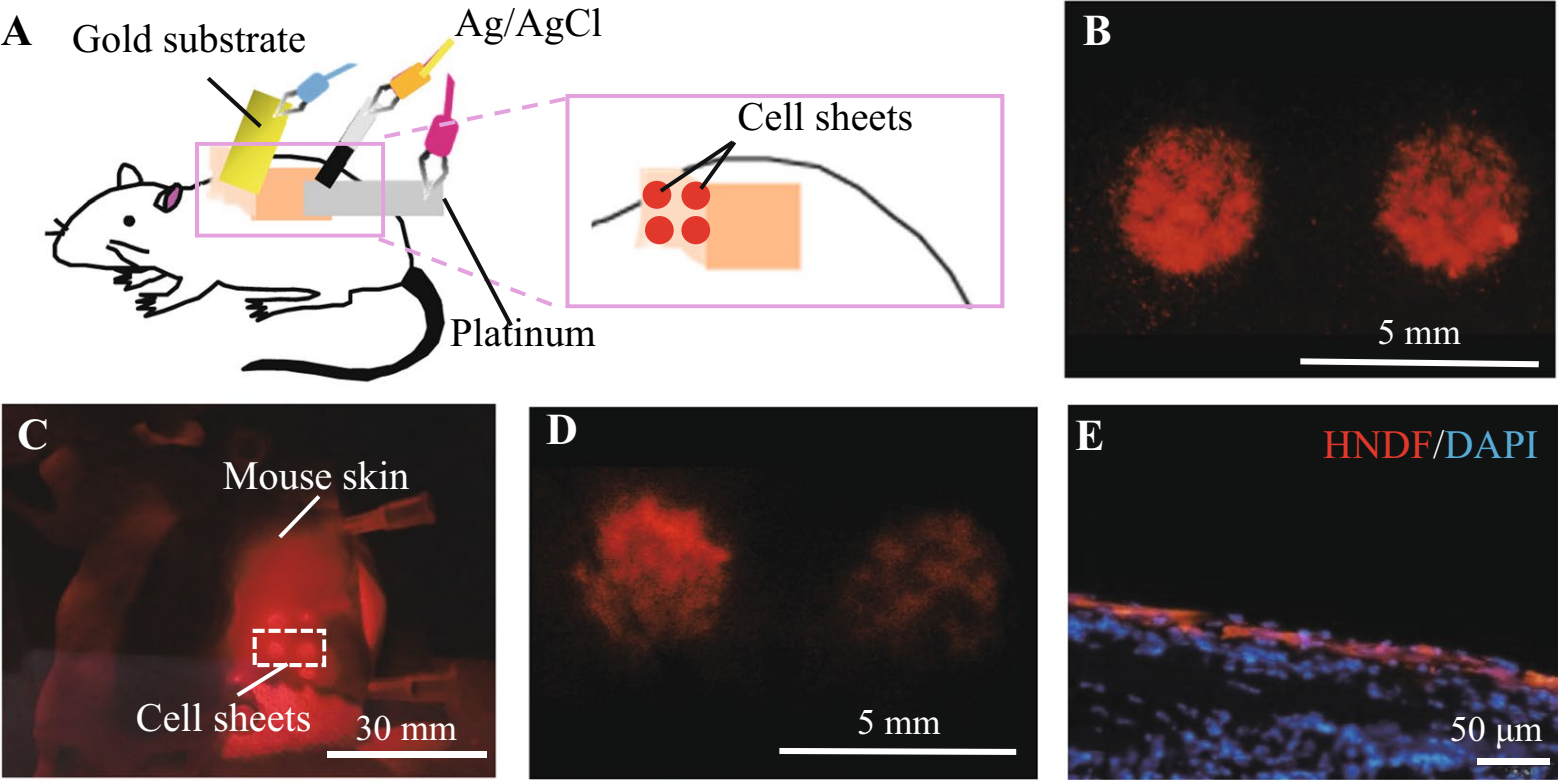
Electrochemical cell sheet transplantation to mice. (**A**) Schematics. (**B**,**C**) Patterned cell sheets on the flat gold substrate modified with oligopeptides (**B**) were transplanted on the subcutaneous pocket (**C**). (**D**) Magnified view of the square region in (**C**). (**E**) Skin at the transplanted site was sectioned and counter-stained with DAPI.

The ability to transplant cell sheets directly from the gold-plated substrate to the body could be beneficial for the treatment of postoperative adhesion and occlusion. To examine direct transplantation, circular-patterned cell sheets (φ3 mm) were prepared on the gold-coated flat substrate and directly transferred to the subcutaneous pocket on the dorsal skin of mice using *in situ* potential application for 5 min (Fig. 4A). The cell sheets on the gold surface (Fig. 4B) were detached and transplanted while maintaining their circular shape (Fig. 4C,D). At 24 h post-transplantation, the skin sample, including the transplanted cell sheets, was excised from the mouse and stained with DAPI (Fig. 4E), indicating that cell sheets were successfully transplanted into the body.

### Fabrication of tailor-made cell sheets using 3D molds

**Figure 5:**
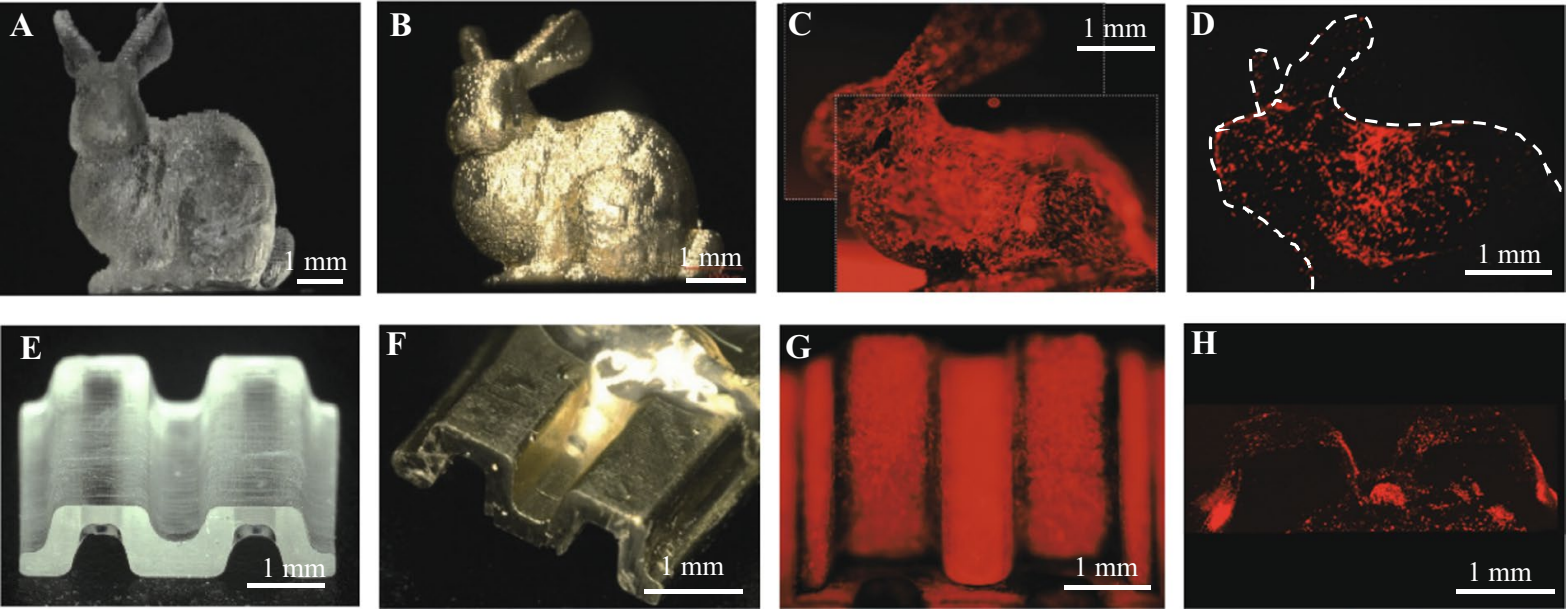
Cell sheets preparation on 3D molds and transfer using electrochemical cell detachment. (**A**,**E**) Bunny (**A**) and small intestine (**E**) molds were fabricated using micro-stereolithography. (**B**,**F**) The molds were coated with gold by electroless plating. (**C**,**G**) RFP-HNDFs were seeded on the molds. The image (**C**) was composed of two merged images indicated with white dashed squires. (**D**,**H**) The cell sheets were transferred to collagen gel using electrochemical cell detachment.

Several 3D molds, including a bunny, small intestine, and needle array, were fabricated using a lab-made microstereolithography system (Fig. 5, Suppl. Fig. 1). The fabrication times were relatively short (20 min for Fig. 5A and 38 min for Fig. 5B). The tips of the tapered needles in Suppl. Fig. 1B were formed down to ~5 μm. The surface of the 3D structures was fully coated with gold using electroless plating (two representative examples are shown in Fig. 5B,F). After the gold surface was modified with oligopeptide, RFP-fibroblasts were seeded and cultured until the surface was entirely covered (Fig. 5C,G). As the feature sizes of the fabricated structures were significantly larger than the cell sizes, there was not much difference in cell behavior on the surface compared to a flat substrate and the cells eventually covered the entire surface. The cell sheets on the 3D molds were encapsulated with collagen gel and then detached and transferred by applying −1.0 V vs. Ag/AgCl for 5 min (Fig. 5D,H). The transfer of cell sheets seems non-perfect. Because there were differences in the fluorescence intensity (probably due to non-uniform cell layer thickness), methods for cell seeding and subsequent culture should be improved using rotation or circulation culture.

In summary, we demonstrated the fabrication of 3D molds using microstereolithography and the covering of mold surfaces with a gold layer using biocompatible plating. Cells were electrochemically detached from the gold surface prepared through gold-plating. In addition, patterned cell sheets were directly transplanted from a flat gold surface to the subcutaneous pocket on the dorsal skin of mice using *in situ* potential application. Furthermore, cell sheets were transferred from the 3D gold-plated molds to a collagen gel. Our next subject will be the fabrication of 3D molds based on information obtained using magnetic resonance imaging. The transplantation of 3D cell sheets will be applied to diseased sites, providing proof of curative effects on specific tissues using tissue-derived cell types in mid-size or large animal models. Although further studies are necessary, this study demonstrated that our approach may provide a new avenue for curing and alleviating postoperative complications using cell sheets with arbitrary shapes.

## Methods

### Electrolytic and electroless gold plating

We compared two gold-plating approaches: cyanide-based electroplating and non-cyanide electroless plating. Substrates coated with gold through cyanide-based electroplating were kindly provided by Nihon Dento Kougyou Co. Ltd, Japan. Note that this gold plating has been used to coat surgical tools such as forceps, tweezers, and scissors as commercial products. Detailed procedures for non-cyanide electroless plating were reported in our previous study^24^. Briefly, a cycloolefin polymer flat substrate (2.4 cm × 2.4 cm, Zeon corporation, Japan) was treated with UV light (7.5 mW/cm^2^ at 185 nm and 50 mW/cm^2^ at 254 nm, KOLI-300S, Koto Electric Co. Ltd., Japan) for 5 min for nanometer-deep etching. Then, the substrate was immersed in a 1.0-M KOH solution **(**Wako Pure Chemical Industries, Japan) at 50 °C for 5 min and followed by a 10% cleaner conditioner (Rohm and Haas, Japan) at 50 °C for 5 min. Gold catalyst was grafted into the etched plate surface by immersing it into a TPN-Au neutral solution (Au, 2.0 g/L, Matsuda Industrial Co., Japan**)** for 5 min at 50 °C, and an NaBH_4_ solution (2.0 g/L, Tokyo Chemical Industry Co., Japan) for 5 min at 50 °C. Subsequently, the substrate was catalytically coated with a gold layer by immersing it into the plating bath solution (Table 1) for 1 h at 70 °C. Note that gold was solubilized in the plating bath solution as tiopronin-gold(I) complex, but not cyanide. Between all the steps using different solutions, rinsing with pure water was performed at least 10 times.

The gold substrates coated by electrolytic and electroless plating were modified with a self-assembled monolayer of oligopeptide (CGGGKEKEKEKGRGDSP, Scrum, Japan**)**, as previously reported^12^. In the procedure, the substrates were immersed in a 50-μM oligopeptide solution at 4 °C overnight. Then, the peptide-modified substrates were sterilized with 70% ethanol and rinsed with phosphate buffer saline (PBS; Invitrogen, USA) for 5 min three times. Subsequently, the substrates were placed in a 6-well culture plate (BD Biosciences, USA). Red fluorescent protein-expressing human neonatal dermal fibroblasts (RFP-HNDFs; Angio-Proteomie, USA) were seeded at a density of 1.0 × 10^5^ cells/2 mL/well in Dulbecco’s modified Eagle medium (Thermo Fisher, Japan) supplemented with 10% fetal bovine serum (Sigma-Aldrich, Japan) and 1% penicillin/streptomycin (Life Technologies, USA). The cells were cultured for 24 h in a humidified 5% CO_2_ incubator at 37 °C and the change in the number of cells was quantified over 4 d of culture in the substrate by counting and analyzing the cells in fluorescent images using microscopy (IX-71; Olympus, Japan) and ImageJ software. Five fluorescent images were used to quantify the cell number for each time point.

### Quantification of adsorption of oligopeptide on gold surface

To characterize the oligopeptide self-assembled monolayer on the gold-plated surface, QCM (AFFINIX QN; Initium, Japan) was used to quantify the density of the oligopeptides on the gold surfaces. A QCM electrode (Initium, Japan) was coated using non-cyanide electroless gold plating as described above. After cleaning with a piranha solution (H_2_SO_4_:H_2_O_2_, 3:1) and rinsing with double distilled water (Milli-Q Advantage; Millipore, Tokyo, Japan), 5-μL of 50-μM oligopeptide solution was placed on the gold surface and incubated for 1 h at 4 °C. Subsequently, the surface was rinsed with double distilled water and dried under N_2_ gas. The adsorbed oligopeptide was quantified using the changes in the resonance frequency according to the Sauerbrey equation.

### Electrochemical detachment of single cells

RFP-HNDFs (1.0 × 10^5^ cells/2 mL/well) were seeded on the substrate coated using electroless gold plating and modified with oligopeptide. After 1 d of culture, the gold substrate (working electrode), Ag/AgCl electrode (reference electrode), and platinum electrode (counter electrode) were placed in PBS and connected to a potentiostat (HA-151; Hokuto Denko, Japan) and −1.0 V was applied for 1, 2, 3, 4, and 5 min. At each time point, the gold substrate was gently rinsed with PBS and fluorescent images were taken to quantify the remaining cells. A bare gold substrate without oligopeptide modification was used as a control. Cells detached using the 5 min potential application were collected with a pipette and reseeded on a 6-well plate. Subsequently, cell growth was monitored for 5 d. Cells collected with trypsin digestion were used as a control experiment.

### Electrochemical detachment of cell sheets

Normal human fibroblast cells (NB1RGB; Riken Cell Bank, Japan) were seeded on the substrate prepared using electroless gold plating and oligopeptide and cultured for 1 week until they reached confluence. A collagen solution was prepared by mixing Type I collagen (3.0 mg/ml, type I-A; Nitta Gelatin, Japan), Ham’s F12 medium (Nitta Gelatin, Japan), and a reconstitution buffer solution (Nitta Gelatin, Japan) at a volume ratio of 8:1:1 on ice. A few drops of the collagen solution were dropped onto the confluent cell sheet and gelated at 37 °C for 30 min in an incubator. After a potential of −1.0 V vs. Ag/AgCl was applied for 5 min, the hydrogel layer was peeled from the substrate. The viability of the cells transferred onto the hydrogel was evaluated using a live/dead fluorometric assay with fluorescein diacetate (FDA; Wako Pure Chemical Industries, Japan) and ethidium bromide (EB; Wako Pure Chemical Industries, Japan**)**. In this assay, the viable cells showed green cytoplasmic fluorescence, whereas the dead cells showed red nuclear fluorescence due to intercalation of EB^25^. Phase-contrast and fluorescent images were acquired using a fluorescent microscope (IX-71; Olympus, Japan).

### Direct transplantation of cell sheets to mice

All animal experiments were approved by the Animal Care and Use Committee of Yokohama National University (Approval No., Animal-2017-05) and conducted in accordance with the requirements. Seven-week-old KSN/Slc nude mice, from Japan SLC, Inc., were anesthetized with isoflurane, and a subcutaneous pocket was operated and prepared on the dorsal skin^15^. To prepare circular cell sheets on a gold substrate modified with oligopeptide, a mask plate with 3-mm diameter holes was prepared using a laser cutting machine (LaserPro C180; Comnet, Taiwan). The mask plate was then gently placed in contact with the gold substrates and RFP-HNDFs were seeded through the holes at 1.0 × 10^5^ cells/hole. After 1 d of culture, the gold electrode with the circular cell sheets (φ3 mm) and platinum and Ag/AgCl electrodes were inserted into the subcutaneous pocket and a potential of −1.0 V was applied for 5 min to transfer the cell sheets from the gold surface to the mice skin. Macroscopic images of the cell sheets transferred onto the subcutaneous pocket were observed with a fluorescent microscope (Dino-Lite Edge M Fluorescence, AnMo Electronics Corp.). To evaluate the samples after 1 d of transplantation, the mice were anesthetized and the skin, including grafts, were harvested. The samples were fixed with 4% paraformaldehyde overnight at 4 °C and embedded in O.C.T compound (SFJ, Japan), and thin (7 μm) frozen sections were cut and stained with 4′,6-diamino-2-phenylindole (DAPI; Sigma-Aldrich, Japan). The cross-sections were observed under a fluorescence microscope. Four cell sheets were transplanted onto a mouse and the experiment was repeated three times (n = 3).

### Mold fabrication and transfer of tailored cell sheets to hydrogel

We have developed several microstereolithography systems to produce 3D structures ranging from micrometer- to millimeter-scale using single-photon and two-photon polymerization processes^26–28^. To fabricate a 3D mold of several millimeters in size with several micrometer resolutions, we constructed a single-photon microstereolithography system using a blue laser (wavelength: 405 nm; laser power: 100 mW; 06-MLD, Cobolt, Sweden). This fabrication system is based on a constrained surface technique, as shown in Fig. 1A. The blue laser beam is scanned by Galvano scanners (GM-1010, Canon Inc., Japan), and then focused onto the bottom glass window with an objective lens (Numerical Aperture: 0.1). The photocurable resin consisted of dipentaerythritol pentaacrylate (SR399, Sartomer, Japan), 1-wt% diphenyl (2,4,6-trimethylbenzoyl) phosphine oxide (97%) (photoinitiator; Sigma-Aldrich, Japan), and 3 wt% of 2-tert-Butyl-4-methylphenol (99%) (radical inhibitor; Sigma-Aldrich, Japan)^29^. The 3D structures were built in a layer-by-layer process while the laser beam scanned the focal plane according to the cross-sectional shape of the 3D model with the photocurable resin. The lamination thickness for microstereolithography was set to 50 μm to obtain sufficient resolutions in the z-direction. The fabricated 3D structures were washed with SOLIFT (Kuraray, Japan) to remove extra resin. The 3D structures were then coated with gold using non-cyanide electroless plating through the procedures mentioned above (except the UV exposure for etching was 30 s instead of 5 min). The gold surface was modified with oligopeptide, on which RFP-HNDFs were seeded.

To replicate the fabricated 3D structures for multiple experiments, the 3D structures were used as templates and an inverted shape against the original 3D structure was molded with polydimethylsiloxane (PDMS). A PDMS prepolymer solution composed of a silicone elastomer and a curing agent (10:1, Shin-Etsu Silicone, Japan) was poured onto the 3D structures and cured at 80 °C for 30 min. After being peeled from the 3D structures, the PDMS molds were placed in a plastic cup and the photocurable resin was poured in there, and then centrifuged for 1 s at 1,000 rpm to fill in the PDMS molds with resin. The photocurable resin was cured by irradiation with a UV lamp (SP-9, Spot Cure, Ushio, Japan) for 1 min. The 3D structures were replicated with the PDMS mold by repeating the processes. The replicated 3D structures were then coated with gold and modified with oligopeptide as described above. After sterilization with 70% ethanol, RFP-HNDFs were seeded on the constructs at 5.0 × 10^6^ cells/2 mL/well in a 24-well plate (BD Biosciences, USA). The cells were cultured for 3 d until the surface of the constructs were fully covered. The constructs with a cell layer were embedded in a collagen gel for 1 d, and by applying −1.0 V vs. Ag/AgCl for 5 min the cell sheets were transferred to the collagen gel.

## Supplementary information

Supplementary Figure 1
