# Supplementary material for: Tailored cell sheet engineering using microstereolithography and electrochemical cell transfer
Source: Sci Rep. 2019 Jul 18;9:10415. doi: 10.1038/s41598-019-46801-9 (PMC6639316; doi:10.1038/s41598-019-46801-9)
Supplement: Supplementary file 1 — Supplementary Figure 1 [file 41598_2019_46801_MOESM1_ESM.pdf]

Tailored cell sheet engineering using microstereolithography and electrochemical cell transfer

Yuka Kobayashi[1], Christopher E.J. Cordonier[2], Yohei Noda[1], Fuminori Nagase[1], Junko Enomoto[1], Tatsuto Kageyama[1], Hideo Honma[2], Shoji Maruo[1], and Junji Fukuda[1*]

[1]Faculty of Engineering, Yokohama National University, 79-5 Tokiwadai, Hodogaya-ku, Yokohama 240-8501, Japan

[2]Faculty of Engineering, Kanto Gakuin University, 1162-2 Ogikubo, Odawara 250-0022, Japan

Correspondent footnote

*Correspondence to Junji Fukuda, Ph.D.

Faculty of Engineering, Yokohama National University, 79-5 Tokiwadai, Hodogaya-ku, Yokohama 240-8501, Japan. Tel: +81-45-339-4008, Fax: +81-45-339-4008, E-mail address: fukuda@ynu.ac.jp

Short title, 3D cell sheet

Keywords (5–10),

Cell sheet; microstereolithography; oligopeptide; electrochemical cell detachment; cytocompatible gold plating

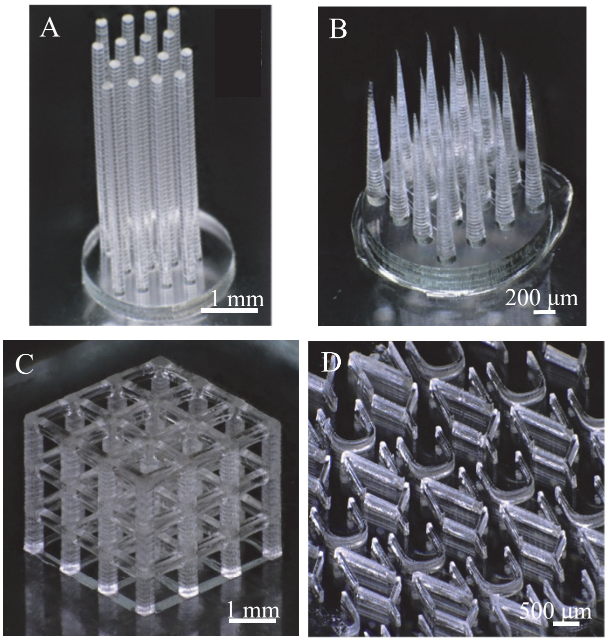
A
1 mm
B
200 µm
C
1 mm
D
500 µm
